# Acceptability of delivery modes for lifestyle advice in a large scale randomised controlled obesity prevention trial

**DOI:** 10.1186/s12889-015-1995-8

**Published:** 2015-07-24

**Authors:** S L Kozica, C B Lombard, D Ilic, S Ng, C L Harrison, H J Teede

**Affiliations:** Monash Centre for Health Research and Implementation (MCHRI), School of Public Health & Preventive Medicine, Monash University, Locked Bag 29, Monash Medical Centre, Clayton, VIC 3168 Australia; Department of Epidemiology and Preventive Medicine, School of Public Health & Preventive Medicine, Monash University, Victoria, Australia; Diabetes and Vascular Medicine Unit, Monash Health, Victoria, Australia

**Keywords:** Evaluation, Lifestyle program, Weight gain prevention, Obesity, Rural, Healthy lifestyle and delivery modes

## Abstract

**Background:**

Preventing obesity is an international health priority and women living in rural communities are at an increased risk of weight gain. Lifestyle programs are needed as part of a comprehensive approach to prevent obesity. Evaluation provides a unique opportunity to investigate and inform improvements in lifestyle program implementation strategies. The Healthy Lifestyle Program for rural women (HeLP-her Rural) is a large scale, cluster randomized control trial, targeting the prevention of weight gain. This program utilises multiple delivery modes for simple lifestyle advice (group sessions, phone coaching, text messages, and an interactive program manual). Here, we describe the acceptability of these various delivery modes.

**Methods:**

A mixed-method process evaluation was undertaken measuring program fidelity, recruitment strategies, dose delivered, program acceptability and contextual factors influencing program implementation. Data collection methodologies included qualitative semi-structured interviews for a sub-group of intervention participants [*n* = 28] via thematic analysis and quantitative methods (program checklists and questionnaires [*n* = 190]) analysed via chi square and t-tests.

**Results:**

We recruited 649 women from 41 rural townships into the HeLP-her Rural program with high levels of program fidelity, dose delivered and acceptability. Participants were from low socioeconomic townships and no differences were detected between socioeconomic characteristics and the number of participants recruited across the towns (*p* = 0.15). A face-to-face group session was the most commonly reported preferred delivery mode for receiving lifestyle advice, followed by text messages and phone coaching. Multiple sub-themes emerged to support the value of group sessions which included: promoting of a sense of belonging, mutual support and a forum to share ideas. The value of various delivery modes was influenced by participant’s various needs and learning styles.

**Conclusion:**

This comprehensive evaluation reveals strong implementation fidelity and high levels of dose delivery. We demonstrate reach to women from relatively low income rural townships and highlight the acceptability of low intensity healthy lifestyle programs with mixed face-to-face and remote delivery modes in this population. Group education sessions were the most highly valued component of the intervention, with at least one face-to-face session critical to successful program implementation. However, lifestyle advice via multiple delivery modes is recommended to optimise program acceptability and ultimately effectiveness.

**Trial registry:**

Australia & New Zealand Clinical Trial Registry. Trial number ACTRN12612000115831, date of registration24/01/2012.

**Electronic supplementary material:**

The online version of this article (doi:10.1186/s12889-015-1995-8) contains supplementary material, which is available to authorized users.

## Background

The global obesity epidemic represents a great public health challenge. The Australian Preventative Taskforce has advocated the need for obesity prevention programs amongst all population groups [1]. Reproductive aged women are an important target group with longitudinal population data revealing high rates of unhealthy weight gain [2] and many barriers to participation in obesity protective behaviours [3]. Furthermore, the prevalence of obesity is elevated in women living in rural settings in comparison to their urban counterparts [4, 5]. Rural communities are often socio-economically disadvantaged, and have relatively poor access to primary health care services, resources and trained health professionals [6]. The need for novel low cost lifestyle programs that can be implemented easily in such groups is critical, where greater program implementation challenges exist. Yet despite this urgency, few healthy lifestyle programs have been implemented in vulnerable target groups such as rural settings [7, 8]. Furthermore, a systematic review highlighted that the efficacy of weight gain prevention programs in rural communities has yet to be established [9].

The International Obesity Task Force highlights the need for monitoring and evaluating all obesity prevention and management programs [10]. In this context, evaluation should focus on the processes required to effectively establish and maintain evidence-based programs in real world conditions [11, 12] to inform policy and practice [13]. Process evaluations through the rigorous documentation and assessment of implementation strategies, improves our understanding of the impact of a program and informs how each program component contributes to outcomes [14]. Process evaluations also assess program internal and external validity, generalisability to diverse populations and identifies factors (program specific and contextual) influencing consistency of program delivery with the protocol [13, 15]. Common components of process evaluation include an assessment of program fidelity (the extent to which the program was implemented as per the protocol), dose delivered (the amount of intended components delivered), context (socio-cultural and physical environment), dose received (the extents to which participants actively engage with, interact with and/or used the program materials) and acceptability (primary and secondary audiences satisfaction with the program) [13, 16, 14].

The value of conducting obesity prevention program evaluations has been established [17]. There has been multiple process evaluations of school based childhood obesity prevention programs conducted [18, 19], demonstrating their value and enabling replication of successful programs to maximise research investments and population benefit [20]. However, there is a current dearth of process evaluations of adult obesity prevention programs, limiting understanding of the interplay between the underlying program theory, processes and outcomes. This information gap also curtails potential for translation of evidence into improved public health outcomes [18, 9]. Further research and evaluation is clearly needed in this area.

Another key research gap is the value and “audience appeal” of obesity prevention programs in women overall and the acceptability of various modes of delivering lifestyle advice (face–to-face and remotely including resources, correspondence and mobile phones) [21]. To our knowledge, there has only been one study which has assessed the acceptability of various delivery modes within an obesity prevention program. In this study the acceptability of delivery modes (group or correspondence delivery) were compared to controls, with significantly less women choosing to participate in face to face groups. However, the group delivery mode produced the largest short-term changes in weight [21]. Thus, further exploration of the acceptability of remote delivery methods alone is warranted.

Understanding participant’s value and preferences for various modes of delivering lifestyle advice, can inform translation and impact on participation rates and program reach, effectiveness and sustainability [21]. In addressing these clear and important research gaps, we aimed to conduct a process evaluation within the context of a large scale rural obesity prevention program, measuring implementation fidelity, dose delivered, context, reach and acceptability of diverse delivery modes. This evaluation focused on informing the acceptability of obesity prevention program implementation strategies in general within complex systems.

## Methods

### HeLP-her Rural program design and theory

The Healthy Lifestyle Program (HeLP-her Rural program) is an integrated community cluster randomised controlled trial (RCT) designed to prevent weight gain in a population of reproductive-aged women living in rural Victorian communities in Australia. Detailed study design methodology are comprehensively explained elsewhere [22]. In summary, the program was designed to be low intensity and focused on participants making small long-term sustainable behaviour changes. In this program 41 rural communities were randomised to intervention or control groups. The control participants attended a single general group health information session. The intervention participants received lifestyle advice through mixed delivery modes including (i) limited personal contact: one group session and (ii) remotely, consisting of: one phone coaching session, monthly text message reminders and a program manual. The intervention content was based on the principles of building self-management informed by the self-determination theory [23] and motivational interviewing [24]. The primary outcome was the difference in weight gain between the control and intervention groups at 12-months.

### Sample size calculations

The sample size calculations were completed a-priori as outlined in the published protocol [22] and clinical trial registry. In the calculation of sample sizes for the primary outcome (weight change over 12-months), adjustments were made for the clustered design prior to program recruitment. The variance inflation factor (VIF) used to achieve this was determined from the average cluster size and the Intra-Cluster Correlation (ICC). The trial was powered to detect a difference of 1.0 kg in weight between groups at 12 months, the weight difference achieved in our original trial (HeLP-her) [25] and the estimated population weight gain [3]. The actual ICC calculated in the previous HeLP-her study was −0.02 using Generalised Estimating Equations, (GEE) [26], despite the negative value is assumed to be equivalent to zero. However we assumed some clustering in this setting although likely small and notably there was little published data to inform the ICC estimates in rural communities. Therefore, to detect an absolute difference in weight between groups, 196 women per intervention arm (control and intervention) were required to participate in the HeLP-her Rural program. Adjusting for the cluster design with a variance inflation factor (VIF) =1.28, cluster size of 15, and allowing for 20 % attrition over 1-year, we aimed to recruit a minimum of 600 women into 40 clusters of 15 women. To allow for inadvertent recruitment challenges 42 towns (clusters) were randomized.

### HeLP-her rural program recruitment

**Fig. 1:**
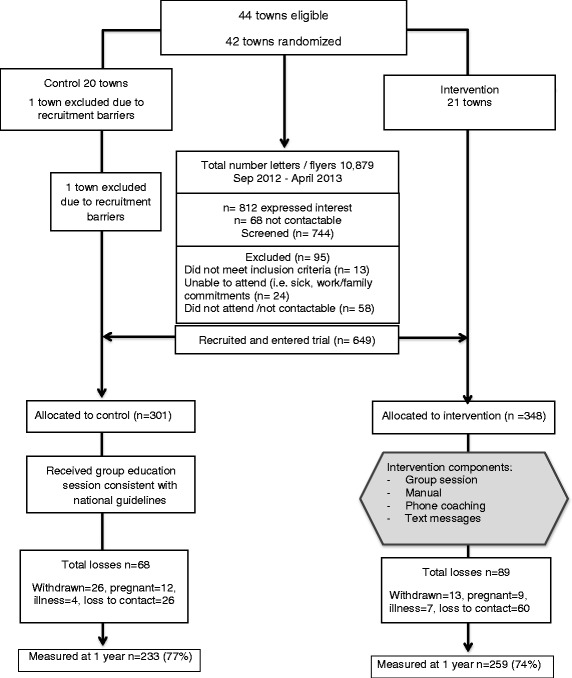
Consort Diagram

Participant recruitment commenced in September 2012 and was completed in April 2013 (Fig. 1). Program recruitment was underpinned by a comprehensive community communication and engagement plan [22]. Participant recruitment strategies were intentionally simple and low cost to reflect usual practice and centred on community integration within current structures. Participants were recruited through the distribution of an invitation letter and flyer to women, provided through primary schools, pre-schools, child care centres and health services in each township. All women aged 18–50 living within each of the 41 selected townships were invited to participate and to assist program recruitment research staff visited each township to provide information in person to participants.

### HeLP-her Rural program delivery and implementation

Multiple intervention components were utilised based on existing evidence of efficacy and feasibility [25, 27, 28]. The delivery modes were designed to reinforce program messages, appeal to various learning styles and preferences and to maximise cost-effectiveness via delivering lifestyle advice remotely, whilst retaining some personal contact. The group education session provided an opportunity to receive personal contact, improve social support amongst participants and encouraged participants to set appropriate health goals based on personalised priorities. During this group session five simple healthy lifestyle messages related to weight gain prevention were presented by the program facilitator (e.g. try to eat 2 serves of fruit and 5 serves of vegetables per day, reduce soft drink intake and take a brisk walk for at least 30 min on most days of the week). Education was provided on building behavioural self-management capacity including: goal setting, action planning, addressing barriers, problem solving and relapse prevention skills. For example rather than advising women to eliminte takeaway food we focused on how they could prepare a healthy meal for the family, despite their multiple family and women commitments. At the end of the group education session participants had generated lifestyle goals and action plans based on their personal priorities and therefore had developed a personalised weight gain prevention strategy.

The program manual was incorporated to promote participants to self-manage their health behaviours though the completion of self-directed activities. Participant were required to document their health goals within their program manual. The phone coaching utilised client orientated counselling approaches and promoted behaviour change through exploring and resolving ambivalence [29]. Women unable to complete the phone coaching were mailed a summary sheet of the core messages provided. The text messages reinforced the program content and accountability [30]. The primary outcome of HeLP-her Rural program was the difference in weight gain between the control and intervention communities at 12 months post program initiation.

### HeLP-her Rural process evaluation design and theory

The evaluation ran parallel to the HeLP-her Rural program which monitored, documented and assessed program implementation processes [16, 31]. Specific dimensions of the process evaluation measured were; program fidelity, recruitment strategies, dose of the program delivered, program acceptability and contextual factors influencing program implementation. A logic model was developed to monitor the program evaluation, reflecting resources and activities (inputs), and underlying theory and anticipated program outcomes.

The intervention study and the evaluation were approved by the Monash Health Research Ethics Committee for research involving humans, project No.12034B.

### Evaluation data collection methods

The evaluation utilised a mixed method data collection approach, recognising the synergistic benefits of both qualitative and quantitative research methods. Methodologies included qualitative semi-structured interviews with a sub-group of intervention participants and quantitative methods (program specific checklists and questionnaires) (Table 1).

**Table 1:** Summary of the HeLP-her Rural program methodology and data collection time points

Outcome	Measurement	Type of data	0 month	6 month	1 year
Program recruitment and reach	Devised program checklists	Quantitative	*√*		
	Australian Bureau of statistics (Socio-Economic Indexes for Areas)				
Program fidelity, dose delivered and context	Devised program checklists, administrative records, staff observation and feedback.	Quantitative	*√*		
Dose received	Participant interviews	Qualitative		*√*	
Program acceptability	Participant interviews	Qualitative		*√*	
	Program devised satisfaction questionnaire	Quantitative			*√*

**Table 2:** Quantitative results on different delivery modes at 12 months

How helpful were the following HeLP-her Rural program components?				
(*n* = 190)	Mean score ± SD	Not helpful	Moderately	Helpful
Group session	3.6 (0.9)	10.2 %	27.8 %	62.7 %
Text messages	3.5 (1.1)	9.8 %	28.4 %	61.7 %
Phone coaching	3.2 (1.1)	12.9 %	36.4 %	50.9 %
Program manual	3.2 (1.1)	16.1 %	37.8 %	45.9 %

Results presented for intervention participants only in descending order

Mean score on a likert scale of 1-5 ± SD

Results additionally presented as relative frequencies (%)

Four types of process evaluation data were collected here utilising qualitative and quantitative methods. This is comprehensive for this type of program evaluation. Data collection methods used included: 1) administration data, 2) checklists and observations completed by the research team, 3a) surveys and questionnaires completed by participants and 3b) qualitative semi-structured interviews [18].

#### Administrative data and contextual data

To explore program reach and context, data from the Australian Bureau of Statistics (ABS) measuring Socio-Economic Indexes for Areas (SEIFA) of relative disadvantage was used. Potential scores ranged from 1–10 with a lower score indicating a greater level of social disadvantage relating to household total income, education attained and unemployment rates [32]. We categorised scores into four groups: SEIFA score 1–2, SEIFA score 3–4, SEIFA score 5–6 and SEIFA score >7. To determine the relationship between SEIFA indexes and the number of program participants recruited from each township, statistical data analysis was performed in consultation with a biostatistician using SPSS version 19.0 for Window [33]. A two-sided value of 0.05 was considered statistically significant. Comparisons between sub-groups were explored using a one-way ANOVA and post-hoc Scheffe test with SEIFA index the between-subject factor.

Building on this, we explored the relationship between participant’s perceptions of the supportiveness of their environment and SEIFA index of their township. Participant’s perceptions of the supportiveness of their environment measured via a baseline questionnaire, “do you believe your local area takes an active role in promoting healthy lifestyle to women”. Exploration of the relationship between participant’s environment and SEIFA scores were tested using chi-square (categorical data).

#### Program checklists and staff observations

Devised program specific evaluation checklists, research team field notes and staff observations were utilised to evaluate program fidelity, dose delivered and program context. Program checklists were informed by previous literature and pilot tested and modified during the early phases of implementation [34–36]. The checklists documented recruitment strategies used, the time taken to deliver the program sessions, the program activities completed, number of people completing all activities, participant engagement and barriers and enablers to delivering health information. The checklists further included a set of core intervention components that needed to be delivered by the program facilitator during the group education session and phone coaching.

This program was delivered by research staff (health professionals) working in pairs; with one staff member facilitating the group education sessions, with the other observing and recording their observations. The staff member observing the delivery of the group education session provided feedback to the program facilitator, ensuring consistency of program delivery across all communities. Fidelity was addressed by all staff undergoing a one day training workshop, receiving on-going support and the use of standard delivery resources. During training staff were advised of the core elements to be delivered and where they could adapt the program to their local audience.

#### Interviews, surveys and questionnaires completed by participants

##### Surveys and questionnaires: quantitative data

All program participants (control and intervention) were invited to complete validated and devised questionnaires at baseline and 12-months post program enrolment. Questionnaires included items on demographic characteristics, socio-cultural and physical environment, health status (psychological and physical), food intake and physical activity. To assess program satisfaction and acceptability participants completed a program devised satisfaction survey and ranked each intervention component (group session, text, phone coaching and program manual) on a likert scale (1–5), where 1 = not at all helpful to 5 = extremely helpful. To see if there were differences in satisfaction scores across the various program components paired T-tests were conducted. A two-sided value of 0.05 was considered statistically significant. Data was analysed using SPSS version 19.0 for Window [33] with results presented as means and frequencies.

##### Qualitative semi-structured interview sampling, methods and analysis

The acceptability of each program component was assessed through qualitative semi-structured interviews conducted in a sub-group of intervention participants only at six months post intervention commencement. A criteria-based, purposive sampling approach was performed regarding the following criteria; 1) towns allocated to receive the intervention only, 2) local government region (equal representation across all five local government regions involved) and 3) town population size (2000–7500). Twelve towns were eligible for participation in the qualitative sub-study and six were randomly selected for participation. All participants from the six selected communities were invited to participate in the semi-structured interviews.

#### Exclusion criteria for the semi-structured interviews

Participants who did not receive the full intended dose of HeLP-her Rural program (initial group session X 1, phone coaching session X 1 and received both the program manual and SMS text messages) were not eligible for participation in the semi-structured interviews. This was because the focus of the semi-structured interviews was to determine the acceptability of the various HeLP-her Rural program components. Subsequently, control participants were also excluded as they did not receive the various intervention components.

#### Semi-structured interview conduct

All participants were provided with information regarding their involvement in the qualitative semi-structured interviews, prior to participation. Written consent was provided by all participants and a letter and follow–up phone call sent to consenting volunteers. To ensure consistency between all interviews a single researcher conducted all interviews guided by a developed interview guide (Additional file 1). The interview guide focused on 5 broad topics; 1) Motivation for program attendance and program expectations, 2) Impact of the HeLP-her Rural program on the broader community, 3) Behaviours change achieved, 4) Exploration of program engagement and utilisation of the various program components and 5) program satisfaction. The interview questions were pilot tested and reworked throughout the interviews allowing for exploration of new ideas and themes. In this manuscript, the qualitative data presented focused primarily on program satisfaction with other qualitative findings to be published elsewhere. This manuscript also reports on quantitative data relating to program fidelity, recruitment, retention and dose delivered.

Those who agreed to participate were interviewed by phone for 25–50 min. Interviews were conducted until data saturation, determined when no new ideas emerged from the interviews, as per standard methods [37]. All qualitative semi-structured interviews were audio-taped and transcribed verbatim. De-identified transcripts were thematically analysed independently by two investigators. Analysis was conducted prior to knowing whether the intervention had been effective at preventing weight gain. In depth discussions of emerging themes took place before a final iteration of the results was agreed upon between investigators. An independent qualitative researcher was included to counteract the dual role of the researcher delivering and evaluating the program.

## Results

### Quantitative data reported by those completing the intervention

#### Reach and recruitment

Participant recruitment commenced in September 2012 and was completed in April 2013 (Fig. 1). Overall, 649 women were recruited to the RCT in the control (*n* = 301) and intervention group (*n* = 348) with a mean baseline age and BMI of 39.6 ± 6.7 years and 28.8 ± 6.9 kg/m^2^ respectively, with no significant difference between groups. This represented approximately 7–10 % of the eligible target population (Fig. 1). The number of participants recruited was sufficient to meet statistical power calculations, in order to detect a true difference in weight gain between groups at 1-year post study commencement. Based on predefined exclusion criteria, less than 12 % of participants (*n* = 95) were excluded post screening. Reasons for exclusion included pregnancy, breastfeeding, taking weight control medication, diagnosis of a serious physical or psychological condition or were not contactable.

#### Program retention

At 12 months, 259 women completed the intervention arm (75 %) with 89 withdrawals primarily related to pregnancy as well as non-completers. Of these 259 women who completed the intervention, 190 women completed the associated satisfaction evaluation questionnaires at 12 months. At 12 months, 233 control participants remained in the study (77 %) with 68 withdrawals primarily related to pregnancy as well as non-completers.

#### Socioeconomic status of participating towns

**Fig. 2:**
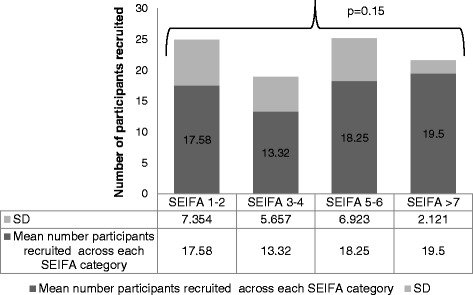
The number of participants recruited into the HeLP-her Rural program according to the socioeconomic index of disadvantage. Legend: This figure indicates the HeLP-her Rural program reach and context, data provided from the Australian Bureau of Statistics (ABS) measuring Socio-Economic Indexes for Areas (SEIFA) of relative disadvantage. Figure 2 reflects the number of participants recruited into the HeLP-her Rural program across based on the townships SEIFA index. Overall, included in the HeLP-her Rural program were 12 townships with a SEIFA index of 1–2; 19 townships with a SEIFA index of 3–4; 8 townships with a SEIFA index of 5–6 and; 2 townships with a SEIFA index of greater than 7. No statistical difference was present between SEIFA indexes and the number of participants recruited from each township (*p* = 0.15)

We reached women from 41 small rural Victorian townships with diverse socioeconomic statuses. SEIFA indexes for relative disadvantage varied between scores of 1–7 (Fig. 2). Overall, included in the HeLP-her Rural program were 12 townships with a SEIFA index of 1–2; 19 townships with a SEIFA index of 3–4; 8 townships with a SEIFA index of 5–6 and; 2 townships with a SEIFA index of greater than 7. The mean number of participants reached in each township was 15.8 ± 6. There was no statistical difference present between SEIFA categories and the number of participants recruited from each township (*p* = 0.15).

In addition, no statistical significant differences existed between SEIFA categories (SEIFA score 1–2, 3–4, 5–6 and >7) and participant’s perceptions of environmental supportiveness for promoting healthy lifestyles (*p* = 0.3).

#### Control group dose delivered

The intended dose for control participants was the attendance of one group education session. Overall, 96 % of the control participants (*n* = 289) attended the group education session.

#### Intervention dose delivered

The intended dose delivered for the HeLP-her Rural intervention arm included: one group education session, receiving a program devised manual, one phone coaching session and monthly text messages. The group education session was attended by 98 % of the intervention participants (*n* = 341). During the group education sessions all participants engaged with the program manual and completed self-management activities with support from the program facilitators. Most group education sessions were delivered within 45–60 min. Phone coaching was completed by 74 % of participants and the average numbers of calls taken to complete phone coaching was 2.84. Text messages were received by 323 intervention participants with 23 participants receiving the same information via mailed post cards, as they reported not having mobile phone. Results on dose received are published elsewhere [38]. In summary, in-depth semi-structured interviews with HeLP-her Rural program participants revealed a high level of program dose received, with almost all interviewees reporting that the program supported them to initiate behaviour change. Factors which influenced behaviour change initiation and continuation included realistic program expectations and the participant’s ability to apply the core program elements including: setting small, achievable behaviour change goals, problem solving and using self-management techniques.

#### Fidelity

Adherence to the Help-Her Rural program protocol [22] was high with all program components delivered as planned to both the control and intervention groups, confirmed through administrative data, completion of program checklists and staff observations. The program checklists completed during the delivery of the group education sessions (intervention arm), confirmed that the five simple program health messages had been discussed amongst the group and that participants had an opportunity to develop and document their personalised health goals. Thus, each participant set a personalised weight gain prevention strategy within their provided program manual.

Furthermore, all program facilitators received specific program training, attending a compulsory one-day training workshop (August 2012). During this workshop the underlying program theory was discussed in depth (self-determination and cognitive behavioural theory) and motivational interview training was provided.

#### Program context

Contextual factors influencing program delivery were documented via researchers completing program devised checklists. The completed checklists indicated that there were a greater number of enablers to program delivery, in comparison to barriers (across control and intervention communities). Observed program contextual enablers included, an interactive and interested group, indicated by participants being actively involved in group discussions and asking numerous questions, evidence of social interactions, friendships, and participant’s encouraging each other during the session. The most frequent barrier to program delivery included reduced group size limiting group discussions and significant background noise made by children present at the sessions, distracting program participants and the program facilitator. However, study design and sample size limited group size in each town and other barriers were addressed by one staff member looking after the children, whilst the other delivered the program to participants.

#### Program acceptability: quantitative results

Program satisfaction was measured quantitatively by questionnaire at 1-year on a likert scale of 1–5 with a higher score indicating greater participant satisfaction. The mean ± SD score (intervention participants only) for the group session (3.6 ± 0.9), text messages (3.5 ± 1.1), phone coaching (3.2 ± 1.1) and program manual (3.2 ± 1.1) Table 2. To assess if there were differences in satisfaction scores across the various program components paired T-tests were conducted. Results revealed that participants reported greater satisfaction with the group session in comparison to the phone coaching (P < 0.00) and the program manual (P < 0.00). Text message satisfaction was higher than the phone coaching (P < 0.00) and the program manual (P < 0.00) with phone coaching scores reduced in comparison to all other components except for the program manual (*P* = 0.63). There was no statistical difference found between satisfaction scores for the group session and text messages (*P* = 0.13).

### Qualitative results

Data saturation for all themes and sub-themes was achieved following 28 participant interviews with a mean age of 39.9 ± 6.2 years and BMI of 28.6 ± 5.2 kg/m^2^. Participants were representative of all intervention communities.

#### Program acceptability: qualitative results

Participants demonstrated a uniform preference for group face-to-face education as the most preferred delivery mode, followed by text messages and phone coaching. The acceptability of the program devised manual’s value varied between intervention participants. Additional supportive quotes can be found in (Additional file 2). Qualitative findings were consistent with our quantitative findings collected at 12 months, supporting the usefulness of the various intervention components and suggesting that face-to-face education was the preferred mode of delivering lifestyle advice.

#### Group education sessions

A recurrent key theme across participant interviews was that the group education session was the most valuable intervention component. Multiple sub-themes emerged to support the value of group sessions, the most prominent was that group sessions promoted a sense of belonging.

*“It made me realise everyone’s trying to do this and you’re not in it alone”*

This peer interaction was described as particularly important amongst women living in isolated rural communities. Participants also explained that group session provided an opportunity for participant’s to share ideas and solutions to overcome barriers to achieving a healthy lifestyle. The sense of group therapy and camaraderie was a motivating factor in initiating lifestyle modifications.

#### Phone coaching and text messages

Numerous sub-themes arose from our data endorsing the benefits of utilising phone coaching within a lifestyle intervention. Participants described that phone coaching provided an opportunity to receive personalised health advice and to resolve questions about the program content. Phone coaching and text messages promoted lifestyle change as they “reminded” participants to self-monitor lifestyle choices and reinforced program messages. Additionally, these initiatives promoted a sense of program “support” and fostered program commitment within participants.

*“It was just a reminder that someone was there wanting to support us if we were struggling”.*

A small number of participants did not view the phone coaching and text messages as valuable. A described drawback of the phone coaching was that the lifestyle advice discussed was “repetitious” and another participant reported “I’m just not good on the phone”. Limitations of text messages were often related to issues with phone reception, “we live in the bush and mobile phone coverage is poor" and issues with text messages arriving at “inappropriate” times. Other participants revealed that the text messages were “easy to ignore”, “delete” and that they “didn’t take enough notice of them”. Some women explain that the text messages were of limited value as they would prefer a more “personalised” approach and favoured “talking to someone”.

#### Program manual

The overarching theme regarding the usefulness of the program manual was that the acceptability varied greatly amongst participants. Approximately half of the program participants interviewed perceived the manual as very “helpful” as it provided informative lifestyle advice that could be “revisited” and “referred back to” and incorporated useful self-management activities such as “setting goal” and problem solving activities.

The majority of women who did not complete the activities, perceived the manual as not being valuable. Emerging sub-themes identified as barriers to greater uptake of the manual included lack of time, multiple commitments, poor literacy skills and the manuals passive written format. However, it was suggested that a strategy to promote better compliance and utilisation of the manual, would be to allocate specific times in sequential group sessions to complete the manual activities.

#### Overall qualitative participant feedback on various program components

The value of utilising multiple delivery modes (face-to-face and remote) resonated strongly with participants. Participant’s agreed that the combination of various program components led to behaviour modifications and improved program acceptability, revealing that “it’s a complete” program and “it all works together”.

*“I think that it’s [all] those little things that get you more motivated, as otherwise you just forget about it and…fall back into your usual routine”.*

However, the value of the group session in combination with other components was a repeated theme.

*“I found the group session brilliant. I got probably the most out of that and then…combination of text messages and phone call”.*

Interviewees also explained that “people have different learning styles”, and “preferences” which influences how they will respond to different delivery modes. Participants emphasised that as “every individual is just so different and different things work for different people” they “wouldn’t remove any” HeLP-her Rural program components in the future.

## Discussion

The evaluation results from the HeLP-her Rural program highlight the acceptability of delivering healthy lifestyle programs via mixed face-to-face and remote delivery modes. We reached women experiencing relative socioeconomic disadvantage and report a high level of program fidelity, dose delivered and program acceptability. The most commonly preferred method of receiving lifestyle advice was via the face-to-face group session. Whilst text messages and phone coaching also had high reported value, they appeared to be most helpful when used in combination with face-to-face contact. Overall, participants emphasised that the combination of various delivery modes maximised program acceptability and value. Based on our evaluation findings, key learnings to optimise the future implementation of the HeLP-her Rural program are described in Table 3.

**Table 3:** The key evaluation learnings to improve the implementation of the HeLP-her Rural program

• Simple low cost participant recruitment strategies were effective in recruiting rural women into a healthy lifestyle program (i.e. the distribution of flyers to women provided through primary schools, pre-schools and health services, media releases and researcher presence in each community). Multiple pathways and repeating recruitment methods may capture those women who are contemplating joining programs.
• High program satisfaction was achieved through combining face-to-face and remote delivery modes.
• Good uptake of phone coaching was achieved within the HeLP-her Rural program through providing flexible session times, scheduling phone coaching time in advance.
• Phone coaching uptake could be improved by research staff clearly explaining to participants the aim and personal benefit of phone coaching at program commencement. Additionally, there is a need to address participants concerns and a need to set realistic outcome expectations prior to phone coaching.
• Program manual use varied greatly with many reasons reported including: lack of time and motivation, forgetfulness, poor literacy levels and personal preferences for more interactive modes of receiving health information. Alternatives to the paper based program manual such as electronic versions or social media forums should be considered where participants might choose the resource that is most relevant to them.

In this healthy lifestyle program, we have engaged women from broad socioeconomic backgrounds including the most socially disadvantaged communities in the State of Victoria, Australia. This is in contrast to previous literature highlighting the challenges of engaging low income population groups into research trials [39], with most weight trials in women attracting highly educated participants of high socio-economic status, who are not representative of the total population [40, 9]. Here we also report no clear relationship between program reach and socioeconomic status of the townships as it appears this locally delivered, community based low intensity program appealed to women of relatively low income backgrounds. This is important, as people experiencing social disadvantage are more likely to be obese, as a result of reduced physical activity participation and poorer diet quality [41, 42]. Potentially, the variation in participant numbers recruited across the 41 townships was influenced by multiple socio-cultural influences such as the presence and engagement of local community champions, social norms, partnerships, program awareness and accessibility [39, 43]. The current results are encouraging and in future greater investigation is needed to identify sociocultural influences and optimise program engagement strategies in low income communities.

Multiple strategies were employed to ensure high program fidelity and dose delivered. We report high program fidelity as result of all program facilitators attending program specific training and standardisation of resources used during program delivery. Dose delivered has been shown to be a limiting factor in intervention success with results from an intensive obesity prevention trial, reporting that only 50 % of participants received the intended program dose [44]. The literature consistently demonstrates low adherence and reduced dose delivered in high intensity face-to-face programs limiting feasibility and effectiveness of intensive programs [28]. We deliberately designed a low intensity intervention program with mixed delivery modes including both face-to-face and remote delivery modes to reduce participant burden and were able to optimise adherence and dose delivered. We also addressed common barriers to attendance and participation which limits dose delivered, such as inconvenient times, childcare and transport by offering multiple delivery times and using local familiar settings such a schools, and allowed toddlers to attend with mothers. We achieved high levels of phone coaching compliance through offering flexible phone coaching times (evening phone coaching conducted), making multiple calls to participants and scheduling phone coaching time in advance strategies which have been used in weight management programs in young women [45]. Our results suggest that program design with some face–to-face content but low participant burden and mixed delivery mode optimises program acceptability of lifestyle obesity prevention programs.

Supporting the value of utilising various delivery modes, two systematic reviews have reported increased program efficacy with combined program delivery modes [46, 8]. Prior to the current study, there was limited evaluation of the acceptability of isolated individual program components such as texts and phone coaching. Most programs include multiple components and do not include process evaluations, making the value of individual components difficult to ascertain [46, 47]. Here we advance the literature by demonstrating that face-to-face delivery, combined with other modes including phone coaching and text messaging are valued by participants. This is consistent with the very few interventions that investigated efficacy of phone coaching and text messaging, which found that these approaches were most efficacious when supported by face-to-face group sessions [48, 46]. This current research affirms the combined delivery modes in the HeLP-her Rural lifestyle program and informs delivery modes for use in future lifestyle interventions. However, greater research exploring the acceptability of various modes of delivering lifestyle advice during formative evaluations and pilot studies is warranted.

Regarding face-to-face delivery, group-based healthy lifestyle programs appear advantageous at the individual and systems level. A systematic review revealed that group-based education sessions produced significantly greater weight management effects over 12 month, compared to individual based treatments [48]. These programs are a resource and cost effective delivery mode for health information and enhance the opportunity for social support [49, 50], as social networks can encourage positive health behaviours, improve self-worth and individual perceptions of control [51]. Furthermore, group education creates a sense of belonging, illustrating to program participants that it is “normal” to struggle to achieve healthy lifestyles. The enhanced sense of belonging promoted by group education is important in rural settings, with increased risks of social isolation [52]. However, as rural women are high users of mobile phones and “remote counselling” has been shown to be efficacious in a recent systematic review [53], combination of group and mobile phone delivery appears ideal [54, 55]. Remote delivery modes also minimise participant burden associated with travel to group sessions [21]. Interestingly, our quantitative findings indicated that phone coaching satisfaction scores were lower than face-to-face group sessions and text messages, highlighting the benefits of utilising diverse delivery methods. Whilst we have demonstrated the benefit of group face-to-face program delivery, exploration of the optimal balances and frequency of supplementary text messages and phone calls is still needed. Additionally, further exploration of program acceptability and methods of delivering lifestyle advice during formative evaluations and pilot studies is also warranted.

### Challenges and lessons learned

The HeLP-her Rural program is one of the largest prevention trials in Australia presenting logistical challenges but providing an opportunity to learn valuable translation and scale-up lessons. To ensure our evaluation would yield useful and reliable results, extensive pre-planning was essential. Vital components included defined evaluation questions, aligning the evaluation with program objectives and prioritising and building an evaluation plan within our program resources (staffing, time, funding and logistics). Table 3 summarises the key evaluation learnings to improve the future implementation of the HeLP-her Rural program.

### Strength and limitations

Strengths of the current study included the application of a mixed-method evaluation approach to a rigorously designed large-scale obesity prevention RCT, targeting a healthy population at risk of weight gain. The use of robust qualitative data analysis methods (theoretical framework and two independent staff conducted analysis) and a range of quantitative data collection tools (administrative data, checklists and questionnaires) strengthened results. Moreover, the purposeful sample utilised in the qualitative data collection increases the generalisability of the results to the wider RCT cohort. We chose to focus on the acceptability of numerous delivery modes for lifestyle advice, as this was a key gap in the literature [20, 21] and can inform the design of future healthy lifestyle programs. We note that the acceptability of the HeLP-her Rural program was not explored in control participants, as they did not receive the active intervention components. However, data pertaining to dose delivered, context and fidelity was recorded for control participants and included within this manuscript. In order to improve program recruitment we believe that more intensive recruitment methods could have increased program uptake, however ethically we were unable to recruit more women than required. Limitations of this study include that our program checklists exploring program fidelity and contextual influences were completed by research staff involved in the trial rather than independent evaluators. In addition fidelity to motivational interviewing theory was not assessed. Exploration of the different delivery modes for healthy lifestyle advice using quantitative and qualitative data were not collected at the same time point.

## Conclusion

Broad scale evaluations of healthy lifestyle obesity prevention programs are not routinely conducted, limiting the ability to maximise on research investments and translate evidence into practice to address the obesity epidemic. Here, results from a comprehensive evaluation of a lifestyle obesity prevention program, demonstrate strong fidelity and high level dose delivery. We reached women from relatively low income rural townships and highlight the acceptability of combined delivery modes including mixed face-to-face and remote delivery modes in this population. Group education sessions were the most highly valued program component and including at least one face-to-face session within a healthy lifestyle program appears integral. Phone coaching and text messages were also valued delivery modes, reinforcing program messages and encouraging commitment to the program. Delivery of lifestyle advice through multiple delivery modes is recommended to optimise program acceptability and ultimately effectiveness.

## Additional files

Additional file 1:
**Participant semi-structured interview schedule.**
Additional file 2:
**Key quotes describing the advantages and disadvantages of the various HeLP-her Rural program intervention components.**
