# Supplementary material for: Acceptability of delivery modes for lifestyle advice in a large scale randomised controlled obesity prevention trial
Source: BMC Public Health. 2015 Jul 24;15:699. doi: 10.1186/s12889-015-1995-8 (PMC4513385; doi:10.1186/s12889-015-1995-8)
Supplement: Additional file 2: — Key quotes describing the advantages and disadvantages of the various HeLP-her Rural program intervention components. [file 12889_2015_1995_MOESM2_ESM.docx]

| **Additional File 2: Key quotes describing the advantages and disadvantages of the various HeLP-her Rural program intervention components** | |
| --- | --- |
| Benefits of the group education session | |
| Sense of belonging | “I just realise there’s so many in the same boat. Like you’re not alone.”  “Especially being isolated out here…it helped me to think that I’m not the only person who struggles.” |
| Learning solutions to overcome barriers from other | Women highlight that hearing other peoples “solutions”, “advice” and “stories” were valuable as these learnings could be applied to their lives. |
| Generate group support and camaraderie | Many women described feeling inspired by the group “camaraderie” and that it made “committing” to the program easier. |
| Enjoyable | The group session was perceived as “fun”, “positive”, “you can have a laugh” |
| Advantages of text messages | |
| Nil reported |  |
| Advantages of text messages | |
| Promoted greater self-awareness in regard to healthy lifestyle choices | “Re-plant the seed, you know, of exercise[ing]…watching what you eat and calories, it’s just a reminder.”  “The more [we] are constantly reminded of things, the more it sets in and becomes a habit” |
| Provided support and accountability | “It felt that they’re (HeLP-her team) really interested in what we’re doing and we’re really going to make that change. “ |
| Encouraged motivation | It provided “motivation” and **“**little inspirational reminder here and there.” |
| Disadvantage of text messages | |
| SMS arrived at inappropriate times | “They would arrive at odd times, I’d kind of glance at it and…it would kind of fade away into the back of my brain” |
| Issue with mobile phone device or reception | “My phone had a meltdown…so I couldn’t go back and refer to them”  “We live in the bush as well and mobile phone coverage is poor" |
| Did not encourage program accountability and motivation | They were “easy to ignore”,  “ I didn’t take enough notice of them”  “I read them but they didn’t really egg me on” |
| Preference for personalised contact | “Talking to someone would be much better than, of there is a text message I will read that later” |
| Advantages of phone coaching | |
| Promoted greater self-awareness | “It keeps everything fresh in your mind what you want to achieve and keep these goals fresh in your mind.” |
| Enhanced program accountability | **“**That’s probably where the phone coaching session was a bit valuable because you’ve got to ‘fess up and talk to a real person and think about why things have or haven’t been working.” |
| Increased motivation | “I got more motivated when I had the personal phone call.” |
| Disadvantage of phone coaching | |
| Unhelpful as repeated previous information provide | “It just seemed to reiterate what we’ve been over and you know I didn’t really find it very helpful” |
| Advantages of the manual | |
| Opportunity to refer back to the health information provided | “Anything that you can refer to on a constant platter is a good thing. The more that you can read about things; the more that you can write things down the more you keep it in your mind, helps you along that road.” |
| Able to refer back to self-management activities | Activities were useful to “keep on track” and to “set goals”.  “Whenever I’d weigh myself maybe and didn’t have the results that I felt I should have got for that week, I would go through and flick through the manual and reset my goals and start again”. |
| Opportunity to track progress | “Tracking my weight once a month. That part was really good, having that so I could track my progress.” |
| Disadvantages of program manual | |
| Time consuming | “I never got round to doing it” and “we don’t have time”. |
| Lacked motivation to complete the manual | Participants explained that **“**the manual was good at the start but then it was easier to put that down and forget about it.” |
| High literacy level required | “I’m not good with written information…information doesn’t sit in well with me if it is written” |
| Preference for visual and interactive learning | “I’m a visual learner”  “It’s more useful to talk to a real person and think about why things have or haven’t been working.” |
